# A Single Ribonucleotide and the Various Possibilities for Charge Transfer Modulation Through ds-DNA: A Density Functional Theory Study

**DOI:** 10.3390/cells15131194

**Published:** 2026-06-30

**Authors:** Boleslaw T. Karwowski

**Affiliations:** DNA Damage Laboratory of the Food Science Department, Faculty of Pharmacy, Medical University of Lodz, ul. Muszynskiego 1, 90-151 Lodz, Poland; boleslaw.karwowski@umed.lodz.pl

**Keywords:** ribonucleotides, ds-DNA, charge transfer, ionisation potential, electron affinity, density functional theory

## Abstract

Ribonucleotides are frequently incorporated into DNA during the replication of genetic information and, if missed during ribonucleotide excision repair, they may undergo phosphodiester bond rearrangement or cleavage. These changes can in turn lead to deformation of the spatial geometry of the local double helix and potentially interfere with charge transfer through ds-DNA. This process is believed to support long-range communication between proteins involved in genome replication and repair. This study theoretically explores how a single embedded riboadenosine (A_3_) affects the structure, electronic properties, and charge-transfer properties of double-stranded DNA ([A_1_G_2_A_3_G_4_A_5_]*[T_5_C_4_T_3_C_2_T_1_]). In particular, the study focuses on four products formed at the ribonucleotide site: native 3′,5′-linkage (R-DNA), the 2′,3′-cyclic phosphate intermediate (IM-R-DNA), rearranged 2′,5-linked (RE-R-DNA), and the single-strand-break cleavage product (SSB-R-DNA). This theoretical investigation was performed at the M06-2X/6-31++G**//M06-2X/D95** level of theory in the aqueous phase. Significant spatial geometry perturbations were found at the central part of ds-oligonucleotides, i.e., the A_3_T_3_|G_4_C_2_ region, where the modified linkage affected the base overlap and stacking interactions most strongly; in the rearranged and cleaved forms, stacking at this site decreased by about 7 kcal•mol^−1^ relative to native DNA. Global electronic analysis showed that R-DNA had the highest ionisation potential and the lowest electron affinity, whereas SSB-R-DNA displayed the lowest adiabatic ionisation potential and the highest adiabatic electron affinity, indicating a much greater tendency to stabilise excess charge. At the base-pair level, G_2_C_4_ was usually the preferred hole sink, except in RE-R-DNA, where G_4_C_2_ was favoured. In contrast, electron localisation was generally favoured at G_4_C_2_, while, in SSB-R-DNA, the A_3_T_3_ pair became the most favourable electron-accepting site. Overall, the results show that even a single ribonucleotide, depending on its linkage chemistry, can substantially reshape charge migration through ds-DNA and may therefore influence lesion recognition, repair efficiency, and genome stability.

## 1. Introduction

The human body is estimated to contain approximately 10^14^ cells. Within each cell can be found the genome—“the seed of life”—located in the nucleus [1]. Deoxyribonucleic acid (DNA) forms a double helix by the complementary pairing of purine:pyrimidine deoxyribonucleosides (dA::T, dG::dC). The stability of their unique sequence guarantees the survival of the species and ensures normal cellular function and development. These processes are exposed to various detrimental external physical and chemical factors, as well as endogenous factors such as reactive oxygen/nitrogen species [2]. These modify nucleosides via hydrogen atom abstraction, hydroxyl radical addition to the unsaturated bonds of base heterocycles, or phosphodiester internucleotide bond cleavage. Any of the above can lead to changes in genetic information, i.e., mutations, and can give rise to changes in genetic information, for example, protein dysfunction [3,4]. The most frequent are single-strand breaks, apurinic/apyrimidinic sites, and 7,8-dihydro-8-oxo-2′-deoxyguanosine (^OXO^dG) [5].

Conversely, a mutation in the genetic code may manifest as a polymerase error during replication. It is important to note that high-fidelity polymerases possess an exonuclease moiety, which acts as a safeguard for normal enzymatic function. However, polymerases can make errors which may, akin to unrepaired DNA damage, result in noncanonical base pairing, such as ^OXO^dG::dA, and consequently induce a mutation in the subsequent replication cycle, such as G → T transversion [6]. To prevent such occurrences, cells have developed various repair mechanisms: nucleotide excision repair (NER), base excision repair (BER), mismatch incision repair (MMR), homologous junction (HJ), non-homologous end joining (NHEJ), and microhomology-mediated repair (MMEJ) [7]. Among these, BER is the primary DNA damage repair mechanism initiated by glycosylases that continuously scan the genome. These enzymes employ electron transfer through the double helix as a nanowire. Proteins such as MutyH, ExoIII, polymerases, and primases possess allosteric iron–sulphur clusters [Fe-S]^2+^ [8]. Moreover, several DNA-processing (Repair and Replication) enzymes employ double-helix-mediated charge transport through the genome. This is known to act as a molecular nanowire, enabling long-range redox signalling and DNA damage detection; however, this activity has not been noted for the aforementioned enzymes (MutYH, Endonuclease III), XPD helicase, DNA primase or replicative polymerases [9,10,11,12].

**Figure 1:**
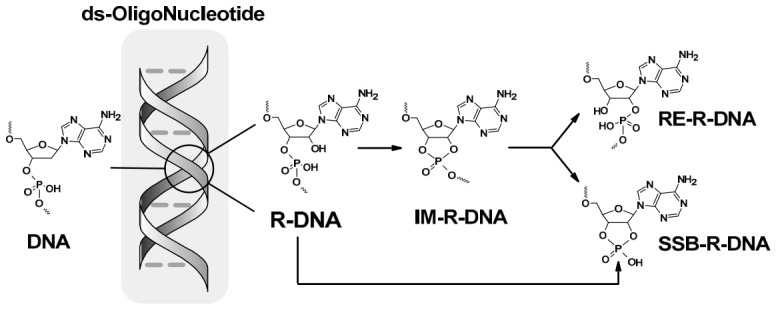
Phosphodiester bond rearrangement of a ribonucleoside moiety, settled in ds-DNA via the Berry pseudorotation mechanism (Ψ) or SN_2_P cleavage, potentially leading to the three different products, i.e., IM-R-DNA, RE-R-DNA and SSB-R-DNA.

This suggests that these crucial enzymes involved in the replication process communicate with each other via electron transfer and scan ds-DNA for the sites of their action [13,14]. The genetic material replication process occurs via lagging- or leading-strand synthesis. The leading strand is synthesised continuously according to 5′→3′ activity of DNA polymerases, which determines the replication fork direction [15,16]. The lagging strand is therefore synthesised discontinuously via Okazaki fragments, which are subsequently fused with the linear DNA strand. This process is initiated by the pol α–primase complex by means of ribonucleotide primer synthesis [17]. The short ribonucleotide fragments are then removed from the growing genome. Imperfections in this process result in ~13,000 ribonucleotides remaining in the yeast genome and 10^6^ in vertebrate cells [18,19]. Additionally, ribonucleoside triphosphates (NTPs) are found in greater abundance in the cell environment than deoxyribose triphosphates (dNTPs) [20]. It has been estimated that, even though the fidelity/selectivity of the polymerase active centre pol ε/δ (replicase) incorporates one NTP per 1250–5000 nucleotides, pol α incorporates one per 625 bases [21,22]. Therefore, during evolution, cells developed a high-performance ribonucleotide excision repair (RER) mechanism which recognises and removes the unwanted ribonucleotide synthons/parts from the genome [23,24,25]. Failure to extract ribonucleoside moieties from the genome can undermine the stability of the double helix by 3′O→2′O phosphodiester bond migration through a thioester intermediate or cause a single-strand break. The above processes arise from the high susceptibility of RNA internucleotide phosphodiester bonds to environmental conditions in comparison to those in DNA [26], as shown in Figure 1.

Taking the above into account, even without any indication of the sequence of nucleobases, the double-helix structure can be affected by the diversity of ribonucleoside–2′-deoxyribonucleoside internucleotide bonds. In light of these factors, this paper is the first theoretical study of how the presence of a ribonucleotide in ds-DNA influences electrons and electron hole migration.

## 2. Materials and Methods

**Figure 2:**
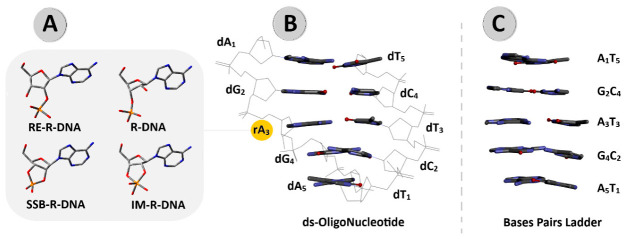
(**A**) Three-dimensional structures of ribonucleotides with different forms of phosphodiester bonds related to the discussed ds-oligonucleotides: R-DNA, RE-R-DNA, IM-R-DNA and SSB-R-DNA; (**B**) the spatial geometry of complete double-stranded oligonucleotide containing adenosine (rA_3_) as the ribonucleoside moiety present in ds-DNA, with the sugar–phosphate backbone indicated by grey lines; (**C**) an example of a base-pair ladder extracted from complete ds-oligo—the charge transfers were subsequently investigated further.

Calculations were carried out as previously outlined and are briefly summarised as follows: the initial structures of short ds-DNAs were built using BioVia Discovery Studio v20.1.0.19295 software [27] and noted as R-DNA d[A_1_G_2_rA_3_G_4_A_5_]*d[T_5_C_4_T_3_C_2_T_1_], RE-R-DNA d[A_1_G_2_rA_3_G_4_A_5_]*d[T_5_C_4_T_3_C_2_T_1_] (2′,5′-phosphodiester linkage between rA_3_ and G_4_), IM-R-DNA d[A_1_G_2_rA_3_G_4_A_5_]*d[T_5_C_4_T_3_C_2_T_1_] (the pentacoordinated species (phosphotriester) between rA_3_ and G_4_), and SSB-R-DNA d[A_1_G_2_rA_3_G_4_A_5_]*d[T_5_C_4_T_3_C_2_T_1_] (rA_3_ containing 2′,3′-cyclic-phosphate). The spatial structures of the modifications are shown in Figure 2. The spatial geometries were optimised as described previously using the QM/MM strategy [28].

The sugar–phosphate backbone from the optimised ds-oligonucleotide structures was removed, leaving a suitable nucleoside pair framework and base-pair systems. Hydrogen atoms were added to saturate the atoms as necessary. The spatial positions of the additional hydrogen atoms added for saturation were optimised at the M06-2x/D95** level of theory.

All discussed energy calculations were conducted in the aqueous phase using density functional theory (DFT) with the M06-2x functional and an augmented polarised valence double-ζ basis set: 6-31++G** in the aqueous phase [29,30]. Tomasi’s polarised continuum model (PCM) was used [31]. This was applied to all discussed ds-oligonucleotides, including complete oligonucleotides, nucleoside pair skeletons without phosphate groups, and base-pair ladders following the extraction of sugar moieties.

The molecular electronic properties discussed here, i.e., vertical/adiabatic ionisation potential (VIP and AIP) and vertical/adiabatic electron affinity (VEA and AEA), were calculated as described previously [32]. The time-dependent DFT (TD-DFT) methodology at the M06-2x/6-31++G** level of theory was used in the aqueous phase for single-point energy calculations of excited states and the transition dipole moment characterisation [32]. The electron coupling (*H_AD_*) was calculated using Generalised Mulliken–Hush methodology [33]. The solvent and solute interaction was analysed in two scenarios—non-equilibrated (**NE**) and equilibrated (**EQ**)—using the polarisable continuum model (**PCM**) as previously described [34].

The charge-transfer rate constants were estimated within the framework of Marcus electron-transfer theory [35]. Based on this framework, the activation free energy (*E_a_*) is determined by the reorganisation energy (λ) and the Gibbs free energy change-driving force (*ΔG*) (Equation (1)):

(1) $$E_{a}=\frac{{\left(\lambda +\Delta_{G}\right)}^{2}}{4\lambda }$$

where λ represents the total reorganisation energy associated with structural relaxation of the donor and acceptor during charge transfer and *ΔG* denotes the driving force of the process.

The charge-transfer rate constant (*k_HT_*) was subsequently calculated using the non-adiabatic Marcus equation [35,36]:

(2) $$k_{HT}=\frac{2\pi }{\hbar }\frac{H_{AD}^{2}}{\sqrt{4\pi \lambda k_{B}T}}\exp\left(-\frac{E_{a}}{k_{B}T}\right)$$

where *H_AD_* is the electronic coupling between the donor and acceptor states, *k_B_* is the Boltzmann constant, T is the temperature, and *ħ* is the reduced Planck constant.

The electronic coupling (*H_AD_*) between donor and acceptor states was evaluated using the Generalised Mulliken–Hush (GMH) approach according to Equation (3) [33]:

(3) $$H_{AD}=\frac{\Delta E_{12}\left|\mu_{12}\right|}{\sqrt{{\left(\mu_{1}-\mu_{2}\right)}^{2}+4\mu_{12}^{2}}}$$

where *ΔE*_12_ is the energy difference between the interacting electronic states of the radical cation and anion. It can be estimated as the difference in energy: *ΔE*_12_ = *E*_HOMO_ − *E*_HOMO−1_ and *ΔE*_12_ = *E*_LUMO_ − *E*_LUMO+1_ (Kohn–Sham orbital method [37,38]). The difference between *μ*_1_ and *μ*_2_, i.e., *μ*_1_ − *μ*_2_, is the difference between the ground state and first excited dipole moment (DM), and *μ*_12_ is the transition DM.

The following energy notation was used as previously: the *E*_geometry_^charge^ of the molecule (neutral form) is described as *E***_0_^0^**, the vertical cation/anion as *E***_0_^+^**/*E***_0_**, the adiabatic cation/anion as *E***_+_^+^**/^+^*E***_−_^−-^**, and the vertical neutral formed from cation/anion state as *E***_0_^+^**/*E***_0_^−^**.

The difference, given in eV, between the mentioned energies corresponds to the suitable electronic states described as follows: **^NE^VIP-** = *E***_0_^+(NE)^** − *E***_0_^0^** (vertical ionisation potential in the NE state); **^EQ^VIP-** = *E***_0_^+(EQ)^** − *E***_0_^0^** (vertical ionisation potential in the EQ state); **AIP** = *E***_+_^+^** − *E***_0_^0^** (adiabatic ionisation potential); ^NE^**VEA** = *E***_0_^–(NE)^** − *E***_0_^0^** (vertical electron affinity in the NE state); ^EQ^**VEA** = *E***_0_^–(EQ)^** − *E***_0_^0^** (vertical electron affinity in the EQ state); and **AEA** = *E***_0_^0^** − *E***_−_^−^** (adiabatic electron affinity). All calculations were performed with the Gaussian G16 (version C.01) software package [39].

The three-dimensional structural analyses of the considered ds-DNAs, based on a standard reference frame, were obtained by a 3DNA software package using the web-based interface w3DNA (web 3DNA) [40].

## 3. Results and Discussion

Although the phosphodiester bond did not play a direct role in the ds-DNA charge transfer process, its migration from the 3′O to the 2′O of ribose through 2′,3′-cyclic phosphate intermediates forced various structural changes in the double helix. As a starting point in this study, the short double-stranded oligonucleotide [A_1_G_2_A_3_G_4_A5]*[T_5_C_4_T_3_C_2_T_1_], denoted as R-DNA, was chosen with riboadenosine (rA) at position A_3_ and a native 3′,5′-internucleotide bond with G_4_. As presented in Figure 1 and Figure 2, the optimised structure of R-DNA was converted into an intermediate product, i.e., IM-R-DNA with a 2′,3′,5′-phosphotriester, which was subsequently converted into RE-R-DNA with a 2′,5′-phosphodiester bond between A_3_ and G_4_ and SSB-R-DNA with an A_3_ terminal 2′,3′-cyclicphosphate and a free 5′OH from the G_4_ nucleotide moiety.

The M06-2X functional was selected due to its well-documented ability to accurately describe noncovalent interactions, particularly π–π stacking interactions, which play a crucial role in charge migration through DNA [30,41]. Furthermore, the same computational protocol (M06-2X/D95** for geometry optimisation followed by M06-2X/6-31++G** single-point calculations) has been successfully applied in previous studies concerning charge transfer through damaged ds-DNA, where it provided chemically meaningful and internally consistent results [41,42]. Therefore, the present study primarily aimed to compare the relative influence of various ribonucleotide-derived phosphodiester modifications on charge migration rather than obtaining absolute charge-transfer parameters. Importantly, all investigated systems were treated consistently at the same level of theory; as a result, the observed trends and relative differences are expected to be significantly less sensitive to the particular choice of functional than the absolute numerical values. Nevertheless, simple benchmarking calculations were performed and are presented in the Supplementary Materials for reference [42]. Following spatial structure optimisation at the M06-2x/D95** level of theory in the aqueous phase, the phosphate groups were removed from the above-mentioned ds-oligonucleotides, leaving only the nucleotide skeleton, which was subsequently converted into the nucleobase pair skeleton (Figure 2B,C). This strategy allowed the influence of the additional hydroxyl group on the double-helix structure, stacking interactions, and charge-transfer rates to be investigated [43].

### 3.1. Influence of Riboadenosine on the ds-DNA Double-Helix Structure

The *π–π* interaction between neighbouring nucleobase pairs is strongly dependent on heterocyclic overlapping (OvL) and the distance between them (*h-Rise*). As shown previously, the migration of the additional charge through ds-DNA results in its settling into the most favourable position. This forces structural relaxation with subsequent adiabatic cation or anion radical formation. A comparison of the anion and cation forms of ds-oligo with the neutral equivalent reveals the following differences, measured as the root mean square deviation (RMSD): SSB-R-DNA > RE-R-DNA > R-DNA > IM-R-DNA and IM-R-DNA > R-DNA > SSB-R-DNA > RE-R-DNA, respectively (Table 1). To quantify the extent of structural reorganisation induced by charge distribution, the root mean square deviation (RMSD) of atomic coordinates was calculated between the optimised neutral and charged structures (adiabatic cation or anion state) according to Equation (4):

(4) $$RMSD=\sqrt{\frac{1}{N}\sum_{i=1}^{N}\left[{\left(x_{i}^{N}-x_{i}^{C}\right)}^{2}+{\left(y_{i}^{N}-y_{i}^{C}\right)}^{2}+{\left(z_{i}^{N}-z_{i}^{C}\right)}^{2}\right]}$$

where: *N* is the number of atoms, *x^N^_i_*, *y^N^_i_*, and *z^N^_i_* are the Cartesian coordinates of the *i* atom in neutral ground geometry, and *x^C^_i_*, *y^C^_i_*, and *z^C^_i_* are the Cartesian coordinates of the *i* atom in cation or anion state of molecules in ground geometry.

The RMSD values therefore provide a quantitative measure of the overall geometrical relaxation accompanying electron loss or adoption. Larger RMSD values indicate more pronounced conformational changes and a greater degree of structural adaptation required for charge accommodation. This is described in more detail in the pioneering work of Kabasch [44,45].

This order was observed in both cases when the complete double helix and base-pair ladder were considered. Additionally, it should be noted that the RMSD value reported for native ds-DNA was twice as low as that calculated for the starting oligo with the ribonucleotide synthon, i.e., R-DNA. Careful analysis of the internal structural double-helix parameters revealed significant decreases in the base pair rA_3_T_3_|G_4_C_2_ heterocycle overlapping, in [Å^2^], of R-DNA (1.33) and RE-R-DNA (1.17) oligonucleotides, in comparison to native ds-DNA (3.73 Å^2^). Additionally, only slight fluctuations were observed in the distance between nucleobase pairs in a range of −0.08 and 0.13 [Å] of all the discussed ds-oligonucleotides in relation to native DNA (Table 1). Electron loss by the system and adiabatic radical anion formation caused significant OvL decreases in the case of rA_3_T_3_|G_4_C_2_ of R-DNA and RE-R-DNA up to 0.63 and 0.22 [Å^2^], respectively, and 1.86 for G_2_C_4_|rA_3_T_3_ of IM-R-DNA, leaving OvL parameter values almost unchanged for SSB-R-DNA. In contrast to the above, the appearance of the extra electron and adiabatic radical anion formation caused a significant decrease in rA_3_T_3_|G_4_C_2_ heterocycle overlapping [Å^2^], with up to 0.86 in the case of SSB-R-DNA, with a subsequent increase in the rise parameters [Å] from 2.85 in the neutral form to 3.43 after the electron settled in the ds-oligo environment.

The structure of the oligonucleotide double helix is stabilised both by the hydrogen bonds between the complementary nucleobases and stacking interactions between the neighbouring base pairs [46]. The latter is sensitive to the structural changes discussed above. In these studies, the stacking interaction energy (*E*_ST_) in kcal•mol^−1^ was calculated at the M06-2x/6-31++G** level of theory in the aqueous phase for the neutral form base-pair ladder and for the nucleoside skeleton in the ground state geometry of the discussed ds-oligonucleotides. Analysis of the obtained data, presented in Table 2, reveals no differences in the R-DNA structure after ribonucleotide insertion compared with unmodified DNA. The *E*_ST_ was measured at 24 kcal•mol^−1^. Internucleotide bond rearrangements (migration or cyclisation) between riboadenosine and the nucleoside attached to its 3′-end 2′-deoxynucleoside led to a significant decrease in the *E_ST_* at the point of formation, i.e., A_3_T_3_|G_4_C_2,_ by approximately 7 kcal•mol^−1^ in comparison with ds-DNA and R-DNA (Table 2).

Furthermore, the stacking interaction energy of the remaining nucleoside pair dimers was almost unchanged for all the ds-oligonucleotides discussed, except for G_4_C_2_|A_5_T_1_ in SSB-R-DNA, for which the *E*_ST_ decreased by 5 kcal•mol^−1^ relative to DNA (Table 3). Removal of the sugar moiety from the above structures, leaving only the base-pair ladder, shows that, in all cases, the *E*_ST_ decreases by approximately 50%. This indicates an unspecified interaction between flexible ribose rings that are not connected by the phosphate backbone. However, the trends in stacking interaction energy remained as previously reported. To clarify the influence of ds-oligo structural changes on the *E*_ST_ forced by a negative or positive charge, vertical neutral ds-oligonucleotides as base-pair ladders derived from adiabatic cationic or anionic states were considered. Visible *E*_ST_, in kcal•mol^−1^, decreases were observed for: a) the A_3_T_3_|G_4_C_2_ base-pair dimer of RE-R-DNA, i.e., 2.17 and 2.28 for cation and anion ground state geometry, respectively; b) G_2_C_4_|A_3_T_3_ by 2.06 (adiabatic cation); and A_3_T_3_|G_4_C_2_ by 1.66 (adiabatic anion) of IM-R-DNA. The single-strand break formation, i.e., SSB-R-DNA, causes an *E*_ST_ increase in the G_2_C_4_|A_3_T_3_ dimer by 3.85 kcal•mol^−1^ extracted from the adiabatic cation structure. Surprisingly, the stacking interaction energy (kcal•mol^−1^) in both cation and anion geometries of R-DNA between the central base pair dimers ranged from 0.14 to −0.66, while, for ds-DNA, it was higher—between 0.4 and 1.3 (Table 2 and Table 3). Hence, the above-discussed results indicate that riboadenosine insertion in the ds-DNA structure renders the double helix more structurally resistant to charge transfer than the native ds-oligo.

### 3.2. Electronic Properties of ds-DNA Containing Riboadenosine

The predisposition of molecules to adopt a positive or negative charge can be estimated by the ionisation potential (IP) or electron affinity (EA) [47]. These parameters can be discussed in vertical or adiabatic modes. Additionally, the solvent–solute interaction warrants investigation and can be described as non-equilibrated (NE) or equilibrated (EQ) vertical IP or EA [48,49]. The parameters mentioned above were calculated at the M06-2x/6-31++G** level of theory in the aqueous phase for: (a) complete ds-oligonucleotides containing a sugar–phosphate backbone and nucleobase-pair ladder; (b) nucleoside-pair skeletons; and (c) base-pair ladders only (Figure 2). The above strategy allowed a comparison of the influence/role of the phosphate linkage, sugar moiety, and 2′-hydroxyl group on “global” electronic parameters. As shown in Table 4 and Table 5, in all discussed cases, the highest AIP and lowest AEA were found for R-DNA, which is in good agreement with the structural parameters and stacking energies discussed above.

In contrast to the above, SSB-R-DNA, where the strand continuity was broken down, had the lowest AIP and highest AEA values. Surprisingly, for the remaining ds-oligonucleotides, the order of ionisation potential and electron affinity is incoherent and depends on the degree of the double helix’s completeness. Nonetheless, it can be seen that removal of the sugar–phosphate backbone from the discussed ds-oligonucleotide structures results in ^NE^VIP and ^EQ^VIP decreasing by 0.09 and 0.07, respectively, and AIP increasing by 0.78 eV. Additionally, for all electron affinities (i.e., ^NE^VEA, ^EQ^VEA, and AEA), after the sugar–phosphate skeleton was removed, the average values were 0.19, 0.09, and 1.29 eV, respectively. A similar trend was observed when only PO_2_ moieties were extracted from double-stranded oligonucleotides, leaving the nucleoside pair ladder only (i.e., average value); ^NE^VIP and ^EQ^VIP decreased by 0.25 eV and 0.08 eV, respectively, while AIP increased by 0.39 eV and decreased by 0.24, 0.24, and 0.5 eV, respectively, for ^NE^VEA, ^EQ^VEA, and AEA (Table 4). All the above results suggest that, in the case of the double helix, the electronic parameters of the sugar–phosphate backbone can influence electronic parameters in an unspecified way, depending on its spatial geometry. It should be noted that the flexibility of the backbone can compensate for the structural changes forced by a modified nucleobase [50,51]. However, 3′O → 2′O internucleotide phosphate bond migration, as shown in Figure 1 and Figure 2, can force a distortion of the mutual nucleobase pair and change the ability of individual base pairs to eject or accept electrons.

Nevertheless, across all three structural models presented in Table 4 and Table 5, AIP and AEA exhibited a strong and consistent inverse correlation (r = −0.82 to −0.97) according to Pearson’s correlation, indicating that reduced ionisation potential is systematically associated with increased electron affinity [52]. This trend remained independent of the structural representation—the ds-oligonucleotide, nucleoside-pair ladder, or base-pair ladder—suggesting that the observed relationship reflects an intrinsic property of the system rather than a model-specific artefact. Therefore, for further studies (i.e., related to charge transfer), only the base-pair ladder need be considered.

The electronic properties of the base pairs, each of which forms the subunit of the discussed ds-oligo, were calculated at the same level of theory as previously (M06-2x/6-31++G** in the aqueous phase). The equilibrated vertical ionisation potential and electron affinity, as well as the adiabatic IP and EA, were considered [53]. Owing to changes in the environment in which the discussed ds-oligonucleotides were submerged, the non-equilibrated solvent–solute interactions were not calculated. Note that in the central part of the double helix formed by the nucleobase ladder, water molecules are absent and the solvation layer interacts only with the sugar–phosphate backbone site; therefore, ^NE^VIP and ^NE^VEA have been omitted from the discussion.

A comparison of the electronic properties between all nucleoside and nucleobase pairs isolated from R-DNA, RE-R-DNA, IM-R-DNA, and SSB-R-DNA reveals the average differences for VIP, AIP, VEA, and AEA as follows: in eV, 0.01, −0.07, 0.03, and 0.12, respectively. For the native ds-oligo, i.e., ds-DNA, the above values were, respectively, 0.004, 0.01, −0.04, and −0.05. Increased disorder was observed in the adiabatic state after structural changes (relaxation) were forced by electron loss or adoption. These results support the choice of base pairs for further comprehensive analysis (Table 6).

The lowest vertical ionisation potential (approximately 6.1 eV) was calculated for the G_2_C_4_ and G_4_C_2_ base pairs, with a difference of 0.01 eV. For SSB-R-DNA, however, G_4_C_2_ had a lower VIP than G_2_C_4_ by 0.08 eV. The relaxation of the ds-oligo geometries after electron loss indicates that the G_2_C_4_ moiety was the preferred position for the adiabatic radical. The lowest AIPs (in eV) were found for R-DNA (6.01), IM-R-DNA (5.82), DNA (5.83), and SSB-R-DNA (5.65). Surprisingly, the phosphodiester bond migration from 3′O to 2′O of adenosine A_3_ leads to an inversion of the aforementioned predisposition; the AIP of the G_4_C_2_ pair was found to be lower than that of G_2_C_4_ − 5.82 eV versus 6.23 eV. This strongly indicates that the internucleotide bond structure influences the spatial geometry of oligonucleotides and, therefore, the charge distribution (which is structure-dependent) [54]. The predisposition of the nucleobase pair settled in ds-oligo to additional electron adoption can be estimated using vertical and adiabatic electron affinity parameters. For all investigated ds-oligonucleotides containing a ribonucleoside moiety, the highest VEA was observed for the G_4_C_2_ base pair and for A_3_T_3_ of SSB-R-DNA, with all cases adopting a value of 1.50 eV. It should be pointed out that, for unmodified DNA, this value was found to be higher by 0.09 eV, which indicates the native ds-oligo’s tendency to adopt an electron.

**Figure 3:**
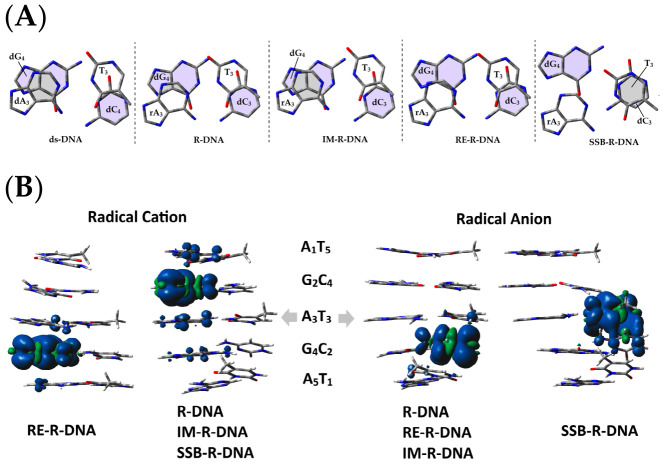
(**A**) A_3_T_3_|G_4_C_2_ base-pair overlapping (shaded area) in ds-DNA, R-DNA, IM-R-DNA, RE-R-DNA and SSB-R-DNA oligonucleotides. (**B**) Graphical representation of the calculated spin density distribution within the nucleobase ladder calculated at the M06-2x/6-31++G** level of theory in the aqueous phase of previously optimised ds-oligo geometries of the adiabatic radical anion and cation forms of the discussed double-stranded oligonucleotides R-DNA, RE-R-DNA, IM-R-DNA, and SSB-R-DNA.

In contrast, a single-strand break alters the VEA pattern in the oligo, as shown in Table 4 and Table 5. The settling of the electron and spatial-geometry relaxation leads to adiabatic radical-anion formation. The calculated AEA parameter (in eV) indicates that G_4_C_2_ is privileged: RE-R-DNA (2.15), DNA (1.99), IM-R-DNA (1.95), and R-RNA (1.86), except for SSB-R-DNA, for which A_3_T_3_ was assigned an AEA value of 2.04 eV. The unique character of SSB-R-DNA stems from the strand discontinuities and structural relaxation at the site of single-strand break formation. This can be explained by the heterocycle overlapping between the reduced [A_3_G_4_]*[T_3_C_2_] dimer (Table 1, Figure 3A) and different spin distributions compared with those found for other ds-oligonucleotides (Figure 3B). Based on the results presented below, it can be concluded that the ribonucleotide subunit appearing in the ds-DNA structure renders the neighbouring part of the oligo less predisposed to charge adoption/stabilisation than native DNA. In contrast to the above, the rearrangement of the internucleotide phosphodiester bond structure, as depicted in Figure 1, forces changes in the electron–hole or excess-electron distribution (variability) (Figure 3).

Therefore, it can be postulated that the presence of a single ribonucleotide in ds-DNA can influence charge migration depending on the linkage structure with the remaining 3′-end oligo strand. Based on spin and charge distribution and inspection of the IPs and EAs of all the nucleobase pairs, the hole and electron transfer in the discussed ds-oligonucleotides can be expected to be disparate (Figure 3).

### 3.3. Charge Transfer Through the Double Helix and the Differences Between R-DNA, RE-R-DNA, IM-R-DNA and SSB-R-DNA

The energy barriers for charge transfer can be estimated in the vertical and adiabatic modes in the aqueous phase at the M06-2x/6-31++G** level of theory. The examined double-stranded pentamers (R-DNA, RE-R-DNA, IM-R-DNA, and SSB-R-DNA) were therefore divided into three trimers, i.e., A_3_G_2_A_3_, G_2_A_3_G_4_, and A_3_G_4_A_5_. According to previous studies [55,56], barriers to hole or electron transfer can be assigned, as shown in Table 5 (the notation has been simplified to the nucleobases of the purine strand), according to an iterative single-step superexchange process. Following the type of the charge migration—vertical or adiabatic—the energies of the donor and acceptor were defined as the sum of the energies of suitable base pairs: charge donor energy A_1_(*E_+_^+^*), G_2_(*E*_0_^0^), and A_3_(*E*_0_^0^), charge acceptor energy A_1_(*E*_0_^0^), G_2_(*E_+_*^0^), and A_3_(*E_+_^+^*) and transfer energy “barrier” assigned in the vertical mode A_1_(*E*_0_^0^), G_2_(*E*_0_*^+^*), and rA_3_(*E*_0_^0^) and in the adiabatic mode A_1_(*E_+_*^0^), G_2_(*E_+_^+^*), and A_3_(*E*_0_^0^) as described previously and denoted here as *ΔG_VB_* and *ΔG_AB_*, respectively [55]. For all ds-oligonucleotides, with the exception of RE-R-DNA, G_2_, which was recognised as a privileged site for hole localisation, lower *ΔG_VB_* and *ΔG_AB_* values were noted for A_3_ → G_2_ migration, i.e., approximately 0.74 and −1.03 kcal, respectively. Meanwhile, in the case of RE-R-DNA, the G_4_C_2_ part was assigned as a radical cation sink, and A_3_ → G_4_ and G_4_ ← A_5_ transfers were favoured (−0.50/−0.93 and −1.24/−1.55 kcal) as in Table 5.

When electron transfer is considered, the G_4_C_2_ base pair shows a predisposition to serve as the settling point of the radical anion in DNA, R-DNA, RE-R-DNA, and IM-R-DNA. The averaged *ΔG* values for the A_3_G_4_ and G_4_A_5_ electron-transfer processes in the vertical/adiabatic modes were identified as follows: −0.10/−0.57 and −0.71/−1.21 kcal, respectively. The situation was different when the oligo strand with rA_3_ was discontinuous after the internucleotide phosphodiester bond was cleaved (i.e., SSB-R-DNA). The A_3_T_3_ base pair next to the single-strand break becomes the sink for the migrating electron through the double helix, as shown in Figure 3B. Moreover, the energetic pattern presented with graphs in Table 7 supports this observation: G_2_ → A_3_ and A_3_ ← G_4_ as well as A_1_ → A_3_ adopted the lowest *ΔG* values in the adiabatic mode: −0.48, −0.68, and −0.56 kcal, respectively. The schematic direction of hole and electron transfer is depicted at the bottom of Table 7. The above results indicate that ds-oligo scanning by proteins involved in replication and repair processes (which utilise electron transfer for communication) depend on the relative positions of the protein and the DNA lesion/modification [57]. These results are in good agreement with previous data, which show that electron transfer can occur between adenines without being significantly affected [58]. Surprisingly, based on these results, it can be proposed that some modifications might be overlooked by different proteins such as glycosylases, because the electronic properties of ribonucleosides depend strongly on the structure of the internucleotide bond (Figure 1 and Figure 2).

### 3.4. The Influence of Riboadenine as Part of ds-DNA on Charge Migration

The internucleotide phosphodiester bond present between the ribonucleoside and the DNA strand at its 3′-end is susceptible to environmental conditions such as temperature and pH [59,60]. Changes in these conditions, as shown in Figure 1, can give rise to different structural outcomes. Even though the base-pair composition remains unmodified, changes in the internucleotide-bond structure influence spatial geometry, base-pair stacking interactions, charge/spin distribution, and the electronic properties of the interactive base pairs that form the double-helix ladder. Therefore, the results discussed above make it possible to predict how a ribonucleotide affects excess-electron and electron–hole transfer through ds-oligo. The charge migration process can be considered in terms of single-step tunnelling, random-walk multistep, and polaron-like hopping [61]. According to Marcus’ theory, the charge (electron or electron–hole) transfer process through the stacked nucleobase-pair ladder can be characterised by a rate constant (*k*_HT_) and the following parameters/energies: *ΔG* (driving force), λ (reorganisation), *E_a_* (activation), and *H_DA_* (electron coupling) [62]. According to Marcus theory, efficient charge transfer is favoured by strong electronic coupling and low activation barriers, whereas increased reorganisation energies or diminished donor–acceptor interactions can significantly decrease the charge-transfer rate. Consequently, structural distortions affecting base stacking and π-orbital overlap can strongly modulate charge migration along the DNA double helix [36]. The larger ***H_AD_*** values indicate stronger donor–acceptor electronic communication and facilitate efficient charge migration through the DNA double helix.

To remain consistent with previous findings, all the above factors were calculated at the M06-2x/6-31++G** level of theory in the aqueous phase. (For further details, please see reference [55].) Marcus’ theory was followed to investigate the influence of four different forms of internucleotide bonds between the A_3_ and G_4_ nucleotides on electron and electron–hole migration through the base-pair ladder, as shown in Figure 2. Depending on the ds-oligonucleotides discussed, the highest activation energies, in eV, were found for hole transfers between A_3_T_3_|G_4_C_2_ (3.01), G_4_C_2_|A_5_T_1_ (7.91), A_1_T_5_|A_3_T_3_ (3.01), and G_2_C_4_|G_4_C_2_ (7.91) in the case of IM-R-DNA and A_3_T_3_|G_4_C_2_ and G_4_C_2_|A_5_T_1_ (4.46) in the case of SSB-R-DNA.

The above indicates that the intermediate product, i.e., 3′,2′-cyclic phosphate, has a negative effect on cation radical transfer by causing changes in the base-pair ladder geometry. The transfer rate between A_3_T_3_|G_4_C_2_ and G_4_C_2_|A_5_T_1_ of IM-R-DNA slowed down to 7.61 × 10^−36^ and 2.69 × 10^−118^ s^−1^, respectively. This is the lowest value among all discussed ds-oligonucleotides with a ribonucleotide synthon. Moreover, for SSB-R-DNA, the hole transfer through the single-strand break A_3_T_3_|G_4_C_2_ and also the descending G_4_C_2_|A_5_T_1_ base-pair dimer, was also found to be prohibited, with *k_HT_ =* 4.36 × 10^−60^ s^−1^ and *k_HT_* = 3.62 × 10^−60^ s^−1^, respectively. Surprisingly, the presence of a ribonucleotide with an unmodified internucleotide bond (R-DNA) causes a decrease in the hole transfer rate to 1.59 × 10^04^ s^−1^ for A_3_T_3_|G_4_C_2_ and propagates it to the G_4_C_2_|A_5_T_1_ (*k*_HT_ = 1.06 × 10^04^ s^−1^) direction.

However, the energies of the electronic ground states are not constant but vary upon charge localisation and subsequent structural relaxation. Following electron or hole transfer, i.e., charge migration, the donor and acceptor moieties adopt new equilibrium (ground) geometries, thus stabilising or destabilising the corresponding charged states. Consequently, the calculated reorganisation energies (λ) and activation barriers (*E_a_*) may occasionally assume very small, zero, or negative values. Within the Marcus framework, such cases correspond to nearly activationless or barrierless charge transfer processes and indicate that the relaxed charged state is energetically more favourable than the initial configuration. Therefore, negative values should be interpreted as a consequence of substantial structural relaxation and charge-induced stabilisation rather than unphysical quantities.

Furthermore, the calculated driving force of base-pair dimers (*ΔG*) adopted values ranging from −0.53 to −0.93 eV, which located the hole transfer process through the discussed ds-oligo in the Marcus normal region (Table 8). For electron transfer, the *E_a_* values were much lower than those for hole transfer, ranging from around 0 to 0.17 eV. Meanwhile, the *ΔG* for the analysed systems adopted a value in the range of −0.05 to −0.76 eV in the case of RE-R-DNA between the A_1_T_5_ and G_2_C_4_ base pairs. For all the mentioned parameters, the electron transfer *k*_HT_ between stacked base pairs of the double helix ranged from 10^−13^ to 10^−14^ s^−1^, except for the A_1_T_5_|G_2_C_4_ system of RE-R-DNA, which was 2.97 × 10^−10^ s^−1^ (Table 8).

It should be emphasised that, in these studies, the calculated charge-transfer rate constants should be interpreted as relative rather than absolute. Experimental studies on DNA-mediated charge transport typically report effective hole-transfer rates in the range of 10^6^–10^9^s^−1^ depending on sequence, distance, and experimental conditions. These processes are substantially slower, however, when charge migration is hindered by structural perturbations or poor electronic coupling [9,63,64,65,66]. In the present study, exceptionally small rate constants were predicted for several transfer pathways, particularly for RE-R-DNA and SSB-R-DNA. These low values originate primarily from diminished electronic coupling (*H_AD_*) and increased reorganisation energies resulting from severe disruption of base stacking and donor–acceptor orbital overlap. Hence, the calculated rates indicate that charge migration is strongly suppressed, but not entirely prohibited, through these pathways. The observed trends therefore suggest that ribonucleotide phosphodiester bond rearrangement and strand cleavage may substantially impede long-range DNA-mediated charge transport.

## 4. Conclusions

Although the genome is hidden deep within the cell nucleus and surrounded by nuclear and cellular layers separated by vast amounts of cytosol, it is continuously exposed to various extracellular and intracellular factors that exhibit either protective or harmful properties. When the balance shifts towards hazardous elements that gain an advantage over the antioxidant pool, for example, their interaction with DNA can give rise to lesion formation. During evolution, cells developed a DNA damage response system that contains a plethora of high-performance, selective, and sensitive mechanisms. As mentioned, base excision repair is the primary guardian of genetic information stability. Its “scouting” proteins—glycosylases—intensively scan the double helix, looking for modified bases like ^OXO^dG. The high efficiency of these proteins, which are present in low numbers in cells, results from their mutual communication via electron transfer, as put forward by Barton [63]. This has important implications for charge transfer, which can be observed from a distance of approximately 5300 bases (1800 nm) [11]. The efficiency of this process depends on the mutual base-pair position in the double-helix structure. This π–π interaction can be influenced by changes in the heterocyclic structure, as shown in theoretical and experimental studies [67]. The appearance of ^OXO^dG in ds-DNA causes a decrease in the rate of radical cation transfer and therefore stops the charge transfer process through the double helix [68]. In addition to modifying the relationship between nucleoside bases, the structure of the sugar–phosphate backbone is also affected. Therefore, the presence of ribonucleotides in the ds-DNA structure warrants investigation. The 3′,5′ phosphodiester junction between ribonucleotides and 2′-deoxynucleosides is susceptible to environmental conditions, such as pH, solvent, and temperature, any of which can result in 2′,5′ migration via a cyclic phosphotriester intermediate or single-strand break formation with 3′,2′-cyclic phosphate termini. Depending on the form adopted, their impact on the base-pair dimer can lead to different outcomes if not repaired by ribonucleotide excision repair.

In this study, for the first time, the influence of different forms of internucleotide phosphodiester bonds between ribonucleotides and 2′-deoxynucleosides in ds-DNA on double-helix spatial geometry and charge transfer was theoretically considered at the M06-2x/6-31 ++G**/M06-2x/D95** level of theory in the aqueous phase. Moreover, the non-equilibrated and equilibrated solvent–solute interaction modes were considered with reference to global electronic ds-oligo properties.

The obtained results are summarised in the following points:

- The influence on ds-DNA structure was observed mainly in the nucleoside pair dimer formed by ribonucleosides and 3′-end 2′-deoxynucleosides [A_3_G_4_]*[C_2_T_3_], as shown in Table 1. This was particularly evident for heterocycle ring overlapping of the neutral form of the discussed ds-oligonucleotides in comparison with ds-DNA, and becomes more obvious after negative or positive charge adoption. This was well supported by the stacking interaction energies calculated for the base-pair and nucleoside-pair dimer. The differences in *E_ST_* for each ds-oligo indicate the different effect of the modified internucleotide bond on the double-helix geometry.
- An investigation of global electronic properties of R-DNA, RE-R-DNA, IM-R-DNA, and SSB-R-DNA in their complete double-helix form, as well as the nucleoside skeleton or base-pair ladder, revealed the following: (a) the highest ionisation potential and lowest electron affinity for R-DNA with the native internucleotide bond and (b) the lowest ionisation potential and highest electron affinity for SSB-R-DNA, where the internucleotide bond was cleaved, leaving the 3′,2-cyclic phosphate on the rA_3_ moiety and a free 5′-OH on dG_4_. The above results suggest that the ribonucleoside subunit present in ds-DNA is unavailable to enzymes that utilise charge transfer for communication. This is evidenced by the electron–hole and electron rate constant (*k_HT_*).
- Careful analysis of base-pair electronic properties elucidated that the G_2_C_4_ base pair adopted the lowest ionisation potential among all ds-oligonucleotides containing riboadenosine (A_3_), with the exception of RE-R-DNA, for which G_4_C_2_ was assigned. Moreover, the highest electron affinity was found for the G_4_C_2_ base pair of R-DNA, IM-R-DNA, RE-R-DNA and A_3_T_3_ of SSB-R-DNA. This indicates that rearrangement of an internucleotide bond can force double-helix geometry fluctuation and bring about changes in the electronic properties of the single base pair.
- A comparative analysis of the electron–hole *k_HT_* reveals its relationship to spatial geometry fluctuation as a result of a different form of internucleotide phosphodiester between rA_3_ and dG_4_ (Table 8). The lowest rate constants in (s^−1^) were found for A_3_T_3_|G_4_C_2_ and G_4_C_2_|A_5_T_1_ of R-DNA, IM-R-DNA, and SSB-R-DNA, whereas, for RE-RDNA, it was noted as almost unaffected (Table 8). The above observation corresponds well with the highest electron activation energy (*E_a_*) of the previously discussed cases.
- In contrast, it was observed that electron migration through stacked base pairs was almost unaffected for all the discussed ds-oligonucleotides. The *k_HT_* values were found to be in the range of 10^10^–10^15^ (s^−1^) for each base-pair dimer.

Considering the aforementioned factors, it can be hypothesised that a single ribonucleotide moiety has the potential to significantly alter the electronic properties of double-stranded oligonucleotides. Furthermore, variations in the internucleotide bonds between ribonucleotides and 2′-deoxynucleosides can affect the charge transfer along the double helix, thereby potentially disrupting the processes of DNA lesion recognition and repair.

Given the observed increase in ribonucleotide insertion in highly proliferative pathological cells, understanding their role in radio- or chemotherapy is crucial for enhancing the efficacy and safety of such cancer treatments.

## Figures and Tables

**Table 1:** Analysis of structural parameters of the discussed base-pair ladder, i.e., nucleobase-pair heterocycle overlaps in base-pair dimer, in [Å^2^], and the distance between them denoted as Rise, in [Å]. The geometries of the starting double-stranded oligonucleotides were calculated at the M06-2x/D95** level of theory in the aqueous phase. A comparison of geometry differences between neutral, anionic, and cationic forms of the discussed ds-oligonucleotides calculated as the Root Mean Square Deviation (RMSD) of atomic positions of ^(a)^ the complete ds-oligonucleotide and ^(b)^ the nucleobase ladder only. A_3_ indicates adenosine, except for ds-DNA (A_3_: 2′-deoxyadenosine).

Base Pair Dimer	Base-Pair Heterocycles Overlap [Å^2^]					Rise (*h*) [Å]				
	R-DNA	IM-R-DNA	RE-R-DNA	SSB-R-DNA	DNA	R-DNA	IM-R-DNA	RE-R-DNA	SSB-R-DNA	DNA
Neutral Form										
A_1_T_5_|G_2_C_4_	3.67	3.60	3.66	3.49	3.81	3.09	3.02	3.04	2.95	2.96
G_2_C_4_|A_3_T_3_	4.82	4.49	4.07	3.17	3.44	3.19	3.28	3.16	3.17	3.22
A_3_T_3_|G_4_C_2_	1.33	3.31	1.17	2.93	3.73	2.98	2.86	2.94	2.85	2.93
G_4_C_2_|A_5_T_1_	4.01	3.38	4.33	4.70	3.96	2.97	2.90	2.88	3.03	2.95
Radical Cation Form										
A_1_T_5_|G_2_C_4_	4.79	4.60	3.27	4.56	4.43	3.02	3.08	3.02	2.95	2.94
G_2_C_4_|A_3_T_3_	4.46	1.86	4.10	3.04	2.37	3.12	3.17	3.12	3.15	3.00
A_3_T_3_|G_4_C_2_	0.63	3.18	0.22	2.87	3.83	2.84	2.82	2.84	2.84	2.97
G_4_C_2_|A_5_T_1_	4.52	3.78	4.52	4.47	4.01	2.85	2.89	2.85	2.83	2.92
Radical Anion Form										
A_1_T_5_|G_2_C_4_	3.33	3.31	3.44	4.35	3.80	3.07	3.00	3.07	3.03	3.00
G_2_C_4_|A_3_T_3_	5.00	4.16	3.55	3.53	3.82	3.30	3.66	3.30	3.25	3.52
A_3_T_3_|G_4_C_2_	2.24	3.21	2.68	0.86	3.89	2.99	2.82	2.99	3.43	2.87
G_4_C_2_|A_5_T_1_	4.00	3.90	4.35	4.94	4.03	2.79	2.84	2.79	2.69	2.90
Root Mean Square Deviation (RMSD) of atomic positions, in [Å^2^], of the ds-oligonucleotide structures after charge adoption										
Anion vs. Neutral	^(a)^0.37 ^(b)^0.44b	^(a)^0.34 ^(b)^0.34	^(a)^0.48 ^(b)^0.47	^(a)^1.50 ^(b)^1.37	^(a)^0.17 ^(b)^0.16	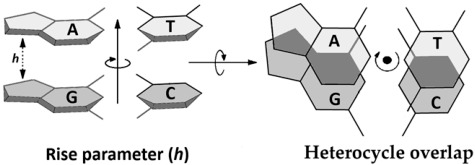				
Cation vs. Neutral	^(a)^0.72 ^(b)^0.69	^(a)^0.88 ^(b)^0.91	^(a)^0.47 ^(b)^0.45	^(a)^0.50 ^(b)^0.48	^(a)^0.36 ^(b)^0.31					

**Table 2:** Stacking interaction energies, *ΔE_ST_*, given in kcal●mol^−1^, between base pairs in dimers of discussed ds-oligonucleotides in adiabatic (Neutr) and vertical neutral forms derived from optimised anion (VN^Anion^) and cation (VN^Cation^) geometries, calculated at the M06-2x/6-31++G** level of theory in the aqueous phase. A_3_ indicates adenosine, apart from ds-DNA (A_3_: 2′-deoxyadenosine). data obtained for base-pair dimer.

Base Pairs Dimer	R-DNA			RE-R-DNA			IM-R-DNA			SSB-R-DNA		
	Neutr	Cation	Anion	Neutr	Cation	Anion	Neutr	Cation	Anion	Neutr	Cation	Anion
A_1_T_5_|G_2_C_4_	14.80	13.02	14.97	15.09	15.17	15.35	15.49	14.42	15.29	14.60	13.74	14.92
G_2_C_4_|A_3_T_3_	13.36	14.00	12.85	14.34	14.69	13.46	13.58	11.52	13.33	14.81	18.66	14.12
A_3_T_3_|G_4_C_2_	13.06	12.92	13.72	13.16	10.99	10.88	14.05	14.69	12.39	12.31	11.88	12.48
G_4_C_2_|A_5_T_1_	15.92	14.94	16.07	15.47	13.91	15.17	15.38	15.83	15.87	15.26	16.19	14.83
A_1_T_5_|A_3_A_5_	0.46	0.51	0.47	0.46	0.51	0.48	0.46	0.49	0.48	0.47	0.56	0.44
G_2_C_4_|G_4_C_2_	0.46	0.48	0.38	0.39	0.41	0.35	0.44	0.53	0.35	0.56	0.56	0.41
A_3_T_3_|A_5_T_1_	0.51	0.53	0.53	0.52	0.51	0.42	0.49	0.54	0.42	0.61	0.63	0.52

**Table 3:** Stacking interaction energies, *ΔE_ST_*, given in kcal●mol^−1^, between nucleoside-pairs in dimers of discussed ds-oligonucleotides in neutral ground state geometries (Neutr), calculated at the M06-2x/6-31++G** level of theory in the aqueous phase. A_3_ indicates adenosine apart from ds-DNA (A_3_: 2′-deoxyadenosine). * data obtained for base-pair dimer.

Nucleoside-Pair Dimer in Neutral Form								
	ds-DNA			ds-DNA	R-DNA	RE-R-DNA	IM-R-DNA	SSB-R-DNA
	Neutr *	Cation *	Anion *					
A_1_T_5_|G_2_C_4_	14.84	14.09	15.87	26.71	27.91	28.12	28.39	27.22
G_2_C_4_|A_3_T_3_	14.60	13.63	14.20	25.29	23.82	25.01	23.46	27.80
A_3_T_3_|G_4_C_2_	14.24	14.75	12.94	24.59	25.12	17.82	18.65	18.32
G_4_C_2_|A_5_T_1_	15.07	15.15	15.44	26.88	27.79	27.53	26.85	22.80

**Table 4:** The electronic parameters in [eV] of the double-stranded oligonucleotides (complete double helix), extracted Nucleosides+Pair ladders and base-pair ladders denoted as DNA, R-DNA, RE-R-DNA, IMR-DNA and SSB-R-DNA, calculated at the M06-2x/6-31++G** level of theory in the aqueous phase. VIP—vertical ionisation potential, AIP—adiabatic ionisation potential, VEA—vertical electron affinity, AEA—adiabatic electron affinity. Non-equilibrated (NE) and equilibrated (EQ) indicate the solvent–solute interaction modes.

ds-Oligonucleotide [28]							Nucleoside-Pair Skeleton					
	^NE^VIP	^EQ^VIP	AIP	^NE^VEA	^EQ^VEA	AEA	^NE^VIP	^EQ^VIP	AIP	^NE^VEA	^EQ^VEA	AEA
DNA	6.72	6.08	5.65	0.84	1.58	2.09	6.68	6.00	5.57	0.71	1.29	1.97
R-DNA	7.03	6.21	7.41	0.73	1.38	1.76	6.23	6.12	6.88	0.56	1.31	1.94
IM-R-DNA	6.91	6.25	4.49	0.68	1.42	3.47	6.87	6.16	5.40	0.57	1.33	2.38
RE-R-DNA	6.84	6.12	3.84	0.62	1.33	4.08	6.70	6.01	5.23	0.49	1.26	3.00
SSB-R-DNA	6.75	6.09	2.76	0.82	1.62	5.55	6.51	6.04	3.00	0.86	1.43	5.17

**Table 5:** The electronic parameters in [eV] of the base-pair ladders denoted as DNA, R-DNA, RE-R-DNA, IMR-DNA and SSB-R-DNA, calculated at the M06-2x/6-31++G** level of theory in the aqueous phase. Notation is given in Table 4.

Base-Pair Ladders						
	^NE^VIP	^EQ^VIP	AIP	^NE^VEA	^EQ^VEA	AEA
DNA	6.47	5.98	5.58	0.60	1.34	1.90
R-DNA	6.87	5.89	6.61	0.55	1.51	1.80
IM-R-DNA	6.82	6.05	5.56	0.56	1.34	1.85
RE-R-DNA	6.81	6.01	5.71	0.53	1.29	1.99
SSB-R-DNA	6.81	5.96	4.59	0.72	1.42	2.94

**Table 6:** Electronic parameters, given in eV, of nucleosides pairs ^(**a**)^ and nucleobase pairs ^(**b**)^ isolated from initial ds-oligonucleotides, i.e., adiabatic and vertical ionisation potential (AIP and VIP) and adiabatic electron affinity (AEA and VEA), calculated at the M06-2x/6-31++G** level of theory in the aqueous phase. * Indicates the nucleoside-pair skeletons in which rA_3_ contains a 3′,2′-cyclicphosphate moiety.

	DNA		R-DNA		RE-R-DNA		IM-R-DNA		SSB-R-DNA	
	AIP	VIP	AIP	VIP	AIP	VIP	AIP	VIP	AIP	VIP
**^(a)^dA_1_T_5_**	6.53	6.57	6.69	6.59	6.59	6.56	6.46	6.56	6.55	6.69
**^(b)^A_1_T_5_**	6.60	6.65	6.82	6.68	6.66	6.65	6.58	6.64	6.39	6.67
**^(a)^dG_2_C_4_**	5.90	6.16	6.05	6.23	6.26	6.20	5.86	6.22	5.62	6.19
**^(b)^G_2_C_4_**	5.83	6.13	6.01	6.19	6.23	6.17	5.82	6.18	5.65	6.11
**^(a)^rA_3_T_3_**	6.64	6.67	7.04	6.69	6.55	6.71	6.44	6.70	6.44	6.79
*^(a)^rA_3_T_3_									5.73	6.83
**^(b)^A_3_T_3_**	6.60	6.65	6.87	6.61	6.75	6.66	6.67	6.63	6.56	6.71
**^(a)^dG_4_dC_2_**	6.14	6.16	6.42	6.20	5.45	6.20	6.14	6.20	5.58	6.04
**^(b)^G_4_C_2_**	6.11	6.13	6.29	6.17	5.82	6.17	6.17	6.17	6.02	6.19
**^(a)^dA_5_T_1_**	6.72	6.76	6.89	6.76	6.70	6.74	6.75	6.79	6.31	6.57
**^(b)^A_5_T_1_**	6.72	6.74	6.85	6.74	6.68	6.72	6.73	6.75	6.55	6.67

	AEA	VEA	AEA	VEA	AEA	VEA	AEA	VEA	AEA	VEA
**^(a)^dA_1_T_5_**	1.42	1.41	1.51	1.50	1.49	1.49	1.47	1.49	1.63	1.61
**^(b)^A_1_T_5_**	1.49	1.48	1.41	1.41	1.41	1.41	1.41	1.41	1.60	1.54
**^(a)^dG_2_dC_4_**	1.47	1.49	1.70	1.54	1.54	1.55	1.54	1.55	1.72	1.67
**^(b)^G_2_C_4_**	1.53	1.56	1.46	1.47	1.48	1.49	1.48	1.49	1.68	1.62
**^(a)^rA_3_T_3_**	1.40	1.40	1.44	1.48	1.61	1.45	1.69	1.48	2.22	1.58
*^(a)^rA_3_T_3_									2.74	1.58
**^(b)^A_3_T_3_**	1.48	1.46	1.37	1.42	1.42	1.38	1.42	1.41	2.04	1.51
**^(a)^dG_4_dC_2_**	1.95	1.52	1.80	1.57	2.51	1.56	2.18	1.57	2.19	1.72
**^(b)^G_4_C_2_**	1.99	1.59	1.86	1.50	2.15	1.48	1.95	1.50	1.76	1.62
**^(a)^dA_5_T_1_**	1.39	1.42	1.34	1.38	1.61	1.38	1.33	1.36	1.65	1.54
**^(b)^A_5_T_1_**	1.35	1.38	1.40	1.43	1.39	1.43	1.39	1.41	1.64	1.58

**Table 7:** Energy barriers (Gibbs energy) in ^(a)^ vertical *ΔG_VB_* and ^(b)^ adiabatic *ΔG_AB_* modes of the hole and electron transfer through double-stranded oligonucleotides given in kcal mol^−1^: R-DNA, RE-R-DNA, IM-R-DNA and SSB-R-DNA, calculated at the M06-2x/6-31++G** level of theory in the aqueous phase. Diagrams of radical cation (Hole, •+) and radical anion (excess electron •−) migration through R-DNA, RE-R-DNA, IM-R-DNA, and SSB-R-DNA, with arrows indicating the directions of possible charge transfer and the dashed line indicating transfers for which the *K_HD_* parameters were unassigned. The shaded areas represent the sites of charge localisation. A_3_ indicates adenosine, except for ds-DNA (A_3_: 2′-deoxyadenosine).

Hole Migration	[A_1_G_2_A_3_]*[T_5_C_4_T_3_]		[G_2_A_3_G_4_]*[C_4_T_3_C_2_]		[A_3_G_4_A_5_]*[T_3_C_2_T_1_]		[A_1_G_2_A_3_G_4_A_5_]*[T_5_C_4_T_3_C_2_T_1_]		
	A_1_ → G_2_	G_2_ ← A_3_	G_2_ ← A_3_	A_3_ → G_4_	A_3_ → G_4_	G_4_ ← A_5_	A_1_ ← A_3_	G_2_ ← G_4_	A_3_ ← A_5_
DNA [44]	−0.52^(a)^	−0.84	−0.85	−0.84	−0.51	−0.60	−0.33	−0.01	−0.09
	−0.76^(b)^	−1.09	−1.10	−0.81	−0.53	−0.60	−0.33	−0.29	−0.07
R–DNA	−0.29	−0.72	−0.91	−0.72	−0.23	−0.56	−0.42	−0.19	−0.33
	−0.62	−1.17	−1.36	−0.87	−0.11	−0.56	−0.55	−0.48	−0.44
RE–R–DNA	−0.47	−0.51	−0.52	−0.50	−0.48	−1.24	−0.04	−0.02	−0.76
	−0.43	−0.53	−0.54	−0.93	−0.83	−1.55	−0.11	0.39	−0.72
IM–R–DNA	−0.46	−0.73	−0.73	−0.73	−0.44	−0.55	−0.27	−0.01	−0.11
	−0.76	−1.14	−1.14	−0.78	−0.44	−0.54	−0.38	−0.36	−0.09
SSB–R–DNA	−0.75	−0.90	−0.71	−0.83	−0.71	−0.44	−0.15	0.11	0.27
	−0.93	−1.21	−1.03	−0.85	−0.88	−0.49	−0.28	−0.18	0.39
Electron Migration									
	A_1_ → G_2_	G_2_ ← A_3_	G_2_ ← A_3_	A_3_ → G_4_	A_3_ → G_4_	G_4_ ← A_5_	A_1_ ← A_3_	G_2_ → G_4_	A_3_ ← A_5_
DNA [44]	−0.08	−0.13	−0.12	−0.15	−0.12	−0.62	−0.05	−0.03	−0.50
	−0.05	−0.10	−0.10	−0.57	−0.55	−1.07	−0.05	−0.47	−0.53
R–DNA	−0.06	−0.05	−0.05	−0.03	−0.03	−0.60	0.01	0.02	−0.57
	−0.05	−0.10	−0.10	−0.45	−0.38	−0.99	−0.05	−0.34	−0.61
RE–R–DNA	−0.06	−0.11	−0.12	−0.11	−0.10	−1.00	−0.05	0.01	−0.89
	−0.06	−0.05	−0.06	−0.73	−0.77	−1.69	0.00	−0.67	−0.93
IM–R–DNA	−0.07	−0.08	−0.09	−0.09	−0.08	−0.60	−0.01	0.00	−0.52
	−0.06	−0.06	−0.07	−0.53	−0.54	−1.07	0.01	−0.46	−0.54
SSB–R–DNA	A_1_ → G_2_	G_2_ → A_3_	G_2_ → A_3_	A_3_ ← G_4_	A_3_ ← G_4_	G_4_ → A_5_	A_1_ → A_3_	G_2_ ← G_4_	A_3_ → A_5_
	−0.27	0.18	−0.01	−0.30	−0.18	−0.39	−0.09	−0.30	−0.21
	−0.27	−0.29	−0.48	−0.68	−0.03	−0.30	−0.56	−0.21	−0.27
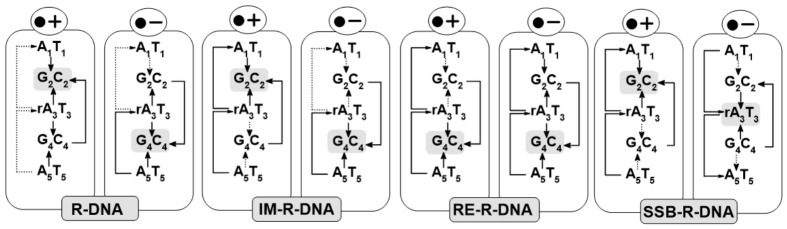									

**Table 8:** Comparison of charge transfer parameters calculated for nucleobase pairs and nucleoside-pair ladders extracted from the optimised structures of R-DNA, RE-R-DNA, IM-R-DNA and SSB-R-DNA double-stranded oligonucleosides. The *ΔG*-driving force [eV], λ-reorganisation energy [eV], *E_a_* activation energy [eV], *H_DA_*-electron coupling, and *k_HT_*-charge rate constant of permissible transfer [s^−1^] were calculated at the M06-2x/6-31++G** level of theory in the aqueous phase.

ds–Oligo	Base–Pair Dimer	Hole Transfer					Electron Transfer				
		*λ*	*ΔG*	*E_a_*	*H_DA_*	*k_HT_*	*λ*	*ΔG*	*E_a_*	*H_DA_*	*k_HT_*
**R–DNA**	A_1_T_5_|G_2_C_4_	0.37	−0.81	0.13	0.26	1.38 × 10^13^	−0.002	−0.05	−0.37	0.02	n.d
	G_2_C_4_|rA_3_T_3_	0.39	−0.86	0.14	0.20	4.07 × 10^12^	0.05	−0.09	0.01	0.03	4.86 × 10^13^
	A_3_T_3_|G_4_C_2_	0.09	−0.59	0.66	0.21	1.59 × 10^04^	0.41	−0.49	0.00	0.04	3.61 × 10^13^
	G_4_C_2_|A_5_T_1_	0.08	−0.57	0.69	0.27	1.06 × 10^04^	0.41	−0.49	0.00	0.04	3.21 × 10^13^
	A_1_T_5_|A_3_T_3_	−0.35	−0.06	−0.12	0.03	n.d.	−0.06	−0.04	−0.04	0.09	n.d.
	G_2_C_4_|G_4_C_2_	0.38	−0.28	0.01	0.01	2.05 × 10^12^	0.37	−0.40	0.00	0.05	6.73 × 10^13^
	A_3_T_3_|A_5_T_1_	−0.32	−0.02	−0.09	0.06	n.d.	0.02	−0.03	0.0001	0.08	6.97 × 10^14^
**RE–R–DNA**	A_1_T_5_|G_2_C_4_	−0.05	−0.43	−1.10	0.27	n.d.	0.01	−0.07	0.17	0.01	2.97 × 10^10^
	G_2_C_4_|A_3_T_3_	−0.04	−0.52	−1.86	0.24	n.d.	0.06	−0.06	0.00	0.04	1.14 × 10^14^
	A_3_T_3_|G_4_C_2_	0.37	−0.93	0.21	0.24	4.70 × 10^11^	0.67	−0.72	0.00	0.07	9.62 × 10^13^
	G_4_C_2_|A_5_T_1_	0.74	−0.87	0.01	0.27	1.15 × 10^15^	0.91	−0.76	0.01	0.03	1.23 × 10^13^
	A_1_T_5_|A_3_T_3_	0.00	−0.09	0.48	0.01	2.26 × 10^05^	0.04	−0.01	0.00	0.09	6.24 × 10^14^
	G_2_C_4_|G_4_C_2_	0.38	−0.41	0.0007	0.002	1.06 × 10^11^	0.67	−0.67	0.00	0.05	5.11 × 10^13^
	A_3_T_3_|A_5_T_1_	0.05	−0.06	0.0004	0.03	6.45 × 10^13^	0.08	−0.03	0.01	0.08	2.77 × 10^14^
**IM–R–DNA**	A_1_T_5_|G_2_C_4_	0.30	−0.77	0.18	0.26	1.63 × 10^12^	−0.0003	−0.07	−0.41	0.01	n.d.
	G_2_C_4_|A_3_T_3_	0.24	−0.86	0.40	0.23	3.54 × 10^8^	−0.01	−0.06	−0.15	0.03	n.d.
	A_3_T_3_|G_4_C_2_	0.02	−0.51	3.01	0.20	7.61 × 10^−36^	0.46	−0.53	0.00	0.06	7.96 × 10^13^
	G_4_C_2_|A_5_T_1_	0.01	−0.56	7.91	0.29	2.69 × 10^−118^	0.49	−0.56	0.00	0.04	3.48 × 10^13^
	A_1_T_5_|A_3_T_3_	0.02	−0.51	3.01	0.20	5.80 × 10^12^	−0.0003	−0.01	−0.11	0.09	n.d.
	G_2_C_4_|G_4_C_2_	0.01	−0.56	7.91	0.29	2.83 × 10^8^	0.46	−0.47	0.00	0.05	6.20 × 10^13^
	A_3_T_3_|A_5_T_1_	0.08	−0.10	0.00	0.01	3.97 × 10^12^	0.04	−0.03	0.00	0.05	1.99 × 10^14^
**SSB–R–DNA**	A_1_T_5_|G_2_C_4_	0.28	−0.74	0.20	0.25	9.85 × 10^11^	−0.13	−0.09	−0.09	0.06	n.d.
	G_2_C_4_|A_3_T_3_	0.27	−0.91	0.38	0.26	8.27 × 10^08^	0.41	−0.36	0.00	0.05	6.15 × 10^13^
	A_3_T_3_|G_4_C_2_	0.02	−0.54	4.46	0.30	4.36 × 10^−60^	0.40	−0.28	0.01	0.11	2.24 × 10^14^
	G_4_C_2_|A_5_T_1_	0.01	−0.53	4.46	0.23	3.62 × 10^−60^	−0.03	−0.12	−0.20	0.02	n.d.
	A_1_T_5_|A_3_T_3_	0.09	−0.17	0.02	0.02	1.13 × 10^13^	0.72	−0.44	0.03	0.04	1.15 × 10^13^
	G_2_C_4_|G_4_C_2_	0.24	−0.37	0.02	0.04	2.62 × 10^13^	0.27	−0.08	0.03	0.02	3.56 × 10^12^
	A_3_T_3_|A_5_T_1_	0.31	−0.01	0.07	0.02	7.54 × 10^11^	0.36	−0.40	0.00	0.04	4.31 × 10^13^

## Data Availability

The data are contained within the article and Supplementary Materials.

## Supplementary Materials

The following supporting information can be downloaded at: https://www.mdpi.com/article/10.3390/cells15131194/s1, Table S1. The energies (in Hartree) of Neural, Vertical Cation (VC^NE^) (NE-non-equilibrated), Vertical Cation (VC^EQ^) (EQ-equilibrated), Vertical Anion (VA^NE^), Vertical Anion (VA^EQ^), Adiabatic Cation (AC), Adiabatic Anion (AA) of complete DNA double helix, nucleoside-pair skeletons and base-pair ladders extracted from ds-oligonucleotides calculated at the M06-2x/6-31++G** level of theory in the aqueous phase. ** the presence of 2′,3′-cyclic phosphate. Table S2. The energies (in Hartree) of Neutral, Vertical Cation (VC^EQ^) (EQ-equilibrated), Vertical Anion (VAEQ), Adiabatic Cation (AC), Adiabatic Anion (AA) and Vertical Neutral from Adiabatic Cation (VNCEQ), Vertical Neutral from Adiabatic Anion (VNA^EQ^) of base pairs extracted from ds-oligonucleotides, calculated at the M06-2x/6-31++G** level of theory in the aqueous phase. Table S3. The energies (in Hartree) of Neutral, Vertical Cation (VC^EQ^) (EQ-equilibrated), Vertical Anion (VAEQ), Adiabatic Cation (AC), Adiabatic Anion (AA) and Vertical Neutral from Adiabatic Cation (VNCEQ), Vertical Neutral from Adiabatic Anion (VNA^EQ^) of nucleosides pairs extracted from ds-oligonucleotides, calculated at the M06-2x/6-31++G** level of theory in the aqueous phase. Table S4. The Energies: Ground (EGR) and Excitation (E^EX^) state energies and Excitation and HOMO Energies, as well as corresponding Dipole Moments Ground, Excitation, and Transition (DM^G^, DM^EX^, D_12_) in Debays of neighbouring base pairs extracted from selected dimers of ds-oligonucleotides, calculated at the M06-2x/6-31++G** level of theory in the aqueous phase using the DFT or TD-DFT methodology. Table S5. Energies: Ground (E^GR^) and Excitation (E^EX^) state energies and Excitation and HOMO Energies as well as corresponding Dipole Moments Ground, Excitation, and Transition (D^MG^, DM^EX^, D_12_) in Debays of distal base pairs extracted from selected trimers of ds-oligonucleotides, calculated at the M06-2x/6-31++G** level of theory in the aqueous phase using the DFT or TD-DFT methodology. Table S6. The energies (in Hartree) of Neutral, Vertical Neutral Cation, Vertical Neutral Anion forms of Base Pairs (BP) extracted from ds-oligonucleotides calculated at the M06-2x/6-31++G** level of theory in the aqueous phase, taken for stacking interaction energy calculations. Table S7. The energies (in Hartree) of Neutral forms of Nucleoside Pairs (NP) extracted from ds-oligonucleotides calculated at the M06-2x/6-31++G** level of theory in the aqueous phase, taken for stacking interaction energy calculations. Zip file (PDB Structures.zip) of the optimised PDB structure of the discussed molecules’ structures.
