# Supplementary material for: A Single Ribonucleotide and the Various Possibilities for Charge Transfer Modulation Through ds-DNA: A Density Functional Theory Study
Source: Cells. 2026 Jun 30;15(13):1194. doi: 10.3390/cells15131194 (PMC13360079; doi:10.3390/cells15131194)
Supplement: Supplementary file 1 [file cells-15-01194-s001.zip › cells-4384523-supplementary.pdf]

Supplementary Materials

A Single Ribonucleotide and the Various Possibilities for Charge Transfer Modulation Through ds-DNA: a Density Functional Theory Study

Boleslaw Karwowski

DNA Damage Laboratory of the Food Science Department, Faculty of Pharmacy, Medical University of Lodz, ul. Muszynskiego 1, 90-151 Lodz, Poland; boleslaw.karwowski@umed.lodz.pl

Table S1. The energies (in Hartree) of Neutral, Vertical Cation (VCNE) (NE-non-equilibrated), Vertical Cation (VCEQ) (EQ-equilibrated), Vertical Anion (VANE), Vertical Anion (VAEQ), Adiabatic Cation (AC), Adiabatic Anion (AA) of complete DNA double helix, nucleosides pairs skeleton and base pairs ladder extracted from ds-oligonucleotides calculated at the M06-2x/6-31++G** level of theory in the aqueous phase. ** the presence of 2′,3′-cyclic phosphate.

	**Neutral**	**VC^{NE}**	**VC^{EQ}**	**AC**	**VA^{NE}**	**VA^{EQ}**	**AA**
	**Complete DNA double helix**						
**DNA**	-12776.34272	-12776.09591	-12776.11927	-12776.13499	-12776.3735	-12776.40072	-12776.41959
**R-DNA**	-12851.59552	-12851.33721	-12851.36736	-12851.32313	-12851.62238	-12851.64638	-12851.66025
**IM-R-DNA**	-12851.53164	-12851.27785	-12851.30183	-12851.36653	-12851.55652	-12851.58389	-12851.65926
**RE-R-DNA**	-12851.52328	-12851.27179	-12851.29856	-12851.38215	-12851.54623	-12851.57216	-12851.67340
**SSB-R-DNA**	-12851.49527	-12851.24718	-12851.27143	-12851.39374	-12851.52553	-12851.55486	-12851.69930
**Nucleosides Pairs extracted from *ds*-oligonucleotides**							
**DNA**	-8846.762064	-8846.516699	-8846.541388	-8846.557304	-8846.788282	-8846.809575	-8846.83449
**R-DNA**	-8921.967684	-8921.738629	-8921.742941	-8921.714808	-8921.988143	-8922.015772	-8922.038826
**IM-R-DNA**	-8921.948716	-8921.696247	-8921.722417	-8921.750244	-8921.969716	-8921.99762	-8922.036012
**RE-R-DNA**	-8921.947024	-8921.700786	-8921.726027	-8921.754777	-8921.965159	-8921.993316	-8922.05722
**SSB-R-DNA**	-8921.912773	-8921.668563	-8921.691966	-8921.7685	-8921.944248	-8921.970681	-8922.050913
**SSB-R-DNA****	-9413.113428	-9412.874148	-9412.891444	-9413.003305	-9413.144983	-9413.165871	-9413.303266
**Base Pairs ladder extracted from *ds*-oligonucleotides**							
**DNA**	-4638.177641	-4637.939729	-4637.957857	-4637.972654	-4638.19955	-4638.226832	-4638.247303
**R-DNA**	-4638.174531	-4637.922081	-4637.957959	-4637.931658	-4638.194566	-4638.229986	-4638.240529
**IM-R-DNA**	-4638.175618	-4637.924849	-4637.953456	-4637.971203	-4638.196335	-4638.224759	-4638.243748
**RE-R-DNA**	-4638.175315	-4637.925119	-4637.954526	-4637.965526	-4638.194711	-4638.22259	-4638.248544
**SSB-R-DNA**	-4638.13748	-4637.88727	-4637.91845	-4637.968742	-4638.164111	-4638.189669	-4638.245390

Table S2. The energies (in Hartree) of Neutral, Vertical Cation (VCEQ) (EQ-equilibrated), Vertical Anion (VAEQ), Adiabatic Cation (AC), Adiabatic Anion (AA) and Vertical Neutral from Adiabatic Cation (VNCEQ), Vertical Neutral from Adiabatic Anion (VNAEQ) of base pairs extracted from ds-oligonucleotides, calculated at the M06-2x/6-31++G** level of theory in the aqueous phase.

	**Neutral**	**VC^{EQ}**	**AC**	**VNC^{EQ}**	**VA^{EQ}**	**AA**	**VNA^{EQ}**
				**DNA**			
**A_1T_5**	-921.191682	-920.947308	-920.949073	-921.191470	-921.243655	-921.243963	-921.191767
**G_2C_4**	-937.252595	-937.027434	-937.038223	-937.240567	-937.307348	-937.306719	-937.251530
**A_3T_3**	-921.192489	-920.948177	-920.949811	-921.192176	-921.243775	-921.244060	-921.192473
**G_4C_2**	-937.252759	-937.027507	-937.028165	-937.252764	-937.308484	-937.324241	-937.233582
**A_5T_1**	-921.191961	-920.944427	-920.945028	-921.192194	-921.244136	-921.243109	-921.190968
				R-DNA			
**A_1T_5**	-921.1915836	-920.9461121	-920.9410189	-921.1845764	-921.2432606	-921.2432878	-921.191514
**G_2C_4**	-937.2523368	-937.0246827	-937.0313976	-937.2342243	-937.3061766	-937.3060275	-937.2520322
**A_3T_3**	-921.1918863	-920.9489492	-920.9392393	-921.1842696	-921.2440937	-921.2421534	-921.1898071
**G_4C_2**	-937.2528971	-937.0260189	-937.0217893	-937.2455681	-937.3081488	-937.3212973	-937.2315082
**A_5T_1**	-921.1919272	-920.9442851	-920.9400522	-921.1852168	-921.2444186	-921.2432019	-921.1909617
				**RE-R-DNA**			
**A_1T_5**	-921.19186	-920.9476602	-920.9471951	-921.1916535	-921.2438448	-921.2437443	-921.1914113
**G_2C_4**	-937.2521336	-937.0255031	-937.0233349	-937.2513896	-937.3067509	-937.306511	-937.2518398
**A_3T_3**	-921.1918473	-920.9469566	-920.9439299	-921.1912442	-921.2424265	-921.2441693	-921.1917338
**G_4C_2**	-937.2523379	-937.025509	-937.0385845	-937.2264774	-937.3068114	-937.3312892	-937.2177109
**A_5T_1**	-921.1922855	-920.9453245	-920.9466747	-921.192437	-921.2446824	-921.243468	-921.1910262
				**IM-R-DNA**			
**A_1T_5**	-921.1918143	-920.9477489	-920.9500207	-921.1916513	-921.2437377	-921.2435141	-921.1914544
**G_2C_4**	-937.2520497	-937.0248902	-937.0381835	-937.2415366	-937.3069455	-937.3064537	-937.2518653
**A_3T_3**	-921.1919974	-920.948423	-920.9466979	-921.1914196	-921.2437721	-921.2441206	-921.1918174
**G_4C_2**	-937.2524119	-937.0256158	-937.0257655	-937.2527988	-937.3074414	-937.3240286	-937.2334281
**A_5T_1**	-921.1916809	-920.9435386	-920.9442832	-921.1919054	-921.2434561	-921.2426209	-921.1904637
				**SSB-R-DNA**			
**A_1T_5**	-921.1847429	-920.9394536	-920.9497511	-921.1915395	-921.2415043	-921.2433989	-921.1916059
**G_2C_4**	-937.245941	-937.0212794	-937.0382787	-937.2417428	-937.3056547	-937.3077906	-937.2503974
**A_3T_3**	-921.1842769	-920.9375391	-920.9431328	-921.1913451	-921.2397354	-921.2592271	-921.1737902
**G_4C_2**	-937.2442531	-937.0166910	-937.0229372	-937.2525556	-937.3036093	-937.3090524	-937.2491161
**A_5T_1**	-921.1852405	-920.9402093	-920.9446189	-921.1920241	-921.2434159	-921.2456259	-921.1916780

Table S3. The energies (in Hartree) of Neutral, Vertical Cation (VCEQ) (EQ-equilibrated), Vertical Anion (VAEQ), Adiabatic Cation (AC), Adiabatic Anion (AA) and Vertical Neutral from Adiabatic Cation (VNCEQ), Vertical Neutral from Adiabatic Anion (VNAEQ) of nucleosides pairs extracted from ds-oligonucleotides, calculated at the M06-2x/6-31++G** level of theory in the aqueous phase.

	**Neutral**	**VC^{EQ}**	**AC**	**VNC^{EQ}**	**VA^{EQ}**	**AA**	**VNA^{EQ}**
		**DNA**					
**A_1T_5**	-1762.893134	-1762.651792	-1762.653012		-1762.947669	-1762.947995	
**G_2C_4**	-1778.958352	-1778.731910	-1778.741430		-1779.015518	-1779.014523	
**A_3T_3**	-1762.898242	-1762.652966	-1762.654058		-1762.951917	-1762.952664	
**G_4C_2**	-1778.957372	-1778.730903	-1778.731789		-1779.015867	-1779.030672	
**A_5T_1**	-1762.892175	-1762.643754	-1762.645196		-1762.942718	-1762.941881	
	R-DNA						
**A_1T_5**	-1762,890521	-1762,648197	-1762,644842	-1762,884923	-1762,945707	-1762,945947	-1762,890844
**G_2C_4**	-1778,957489	-1778,728435	-1778,735096	-1778,938955	-1779,014226	-1779,01979	-1778,962903
**A_3T_3**	-1838,096648	-1837,850796	-1837,83787	-1838,085812	-1838,151121	-1838,149702	-1838,09512
**G_4C_2**	-1778,959669	-1778,731837	-1778,723918	-1778,948846	-1779,017514	-1779,025816	-1778,932405
**A_5T_1**	-1762,893195	-1762,644701	-1762,639974	-1762,885802	-1762,943863	-1762,942612	-1762,891628
	**RE-R-DNA**						
**A_1T_5**	-1762,890753	-1762,649558	-1762,648597	-1762,890054	-1762,945643	-1762,945594	-1762,890228
**G_2C_4**	-1778,956932	-1778,728957	-1778,726772	-1778,956143	-1779,013823	-1779,013646	-1778,956433
**A_3T_3**	-1838,094865	-1837,848327	-1837,854117	-1838,102827	-1838,148058	-1838,154023	-1838,099152
**G_4C_2**	-1778,95191	-1778,724151	-1778,75179	-1778,939217	-1779,009251	-1779,04412	-1778,928014
**A_5T_1**	-1762,893263	-1762,645399	-1762,64713	-1762,893718	-1762,944006	-1762,95244	-1762,900568
	**IM-R-DNA**						
**A_1T_5**	-1762,891187	-1762,650156	-1762,653744	-1762,892057	-1762,94599	-1762,945038	-1762,889878
**G_2C_4**	-1778,956821	-1778,728301	-1778,741349	-1778,945017	-1779,01391	-1779,013398	-1778,956452
**A_3T_3**	-1838,093702	-1837,847647	-1837,856967	-1838,102255	-1838,148042	-1838,155788	-1838,100875
**G_4C_2**	-1778,955869	-1778,727861	-1778,730233	-1778,95832	-1779,013578	-1779,036155	-1778,942036
**A_5T_1**	-1762,892576	-1762,643229	-1762,644659	-1762,893257	-1762,942696	-1762,941585	-1762,890612
	**SSB-R-DNA**						
**A_1T_5**	-1762,884835	-1762,639139	-1762,644268	-1762,892829	-1762,944629	-1762,945556	-1762,890334
**G_2C_4**	-1778,947616	-1778,7236	-1778,744691	-1778,97342	-1779,012446	-1779,014512	-1778,955677
**A_3T_3**	-2329,290362	-2329,039446	-2329,075931	-2329,327469	-2329,34843	-2329,391248	-2329,301387
**G_4C_2**	-1778,951211	-1778,725683	-1778,742418	-1778,946745	-1779,010654	-1779,028193	-1778,962891
**A_5T_1**	-1762,884835	-1762,639139	-1762,644268	-1762,892829	-1762,941272	-1762,945329	-1762,892969

Table S4. The Energies: Ground (EGR) and Excitation (EEX) state energies and Excitation and HOMO Energies, as well as corresponding Dipole Moments Ground, Excitation, and Transition (DMG, DMEX, D12) in Debays of neighbouring base pairs extracted from selected dimers of ds-oligonucleotides, calculated at the M06-2x/6-31++G** level of theory in the aqueous phase using the DFT or TD-DFT methodology.

SYSTEM	B.P. Dimer	E^{GR}	DM^{GR}	E^{EX}	DM^{EX}	D_{12}	E^{HOMO}	E^{HOMO-1}	E^{LUMO}	E^{LUMO+1}
**R-DNA**	$\mathbf{A_1T_5 \mid G_2C_4}$	-3541.892494	7.42	-3541.763322	7.81	3.48	-0.2630	-0.2774	-0.0179	-0.0164
	$\mathbf{G_2C_4 \mid rA_3T_3}$	-3617.09209	10.23	-3616.960274	10.36	5.79	-0.2645	-0.2817	-0.0198	-0.0165
	$\mathbf{rA_3T_3 \mid G_4C_2}$	-3617.096342	13.02	-3616.966144	13.31	6.99	-0.2646	-0.2798	-0.0178	-0.0150
	$\mathbf{G_4C_2 \mid A_5T_1}$	-3541.897146	13.17	-3541.765642	13.18	5.95	-0.2627	-0.2838	-0.0202	-0.0129
**RE-R-DNA**	$\mathbf{A_1T_5 \mid G_2C_4}$	-3541.892498	8.54	-3541.763135	8.96	2.57	-0.2607	-0.2766	-0.0181	-0.0166
	$\mathbf{G_2C_4 \mid rA_3T_3}$	-3617.091646	9.96	-3616.959836	9.92	5.44	-0.2622	-0.2815	-0.0191	-0.0149
	$\mathbf{rA_3T_3 \mid G_4C_2}$	-3617.075178	6.51	-3616.944701	6.75	4.48	-0.2570	-0.2786	-0.0163	-0.0122
	$\mathbf{G_4C_2 \mid A_5T_1}$	-3541.889037	12.38	-3541.757588	12.51	5.89	-0.2622	-0.2830	-0.0191	-0.0124
**IM-R-DNA**	$\mathbf{A_1T_5 \mid G_2C_4}$	-3541.893251	8.43	-3541.763892	8.85	4.15	-0.2611	-0.2763	-0.0183	-0.0166
	$\mathbf{G_2C_4 \mid rA_3T_3}$	-3617.087914	12.74	-3616.956183	12.82	5.96	-0.2633	-0.2819	-0.0195	-0.0159
	$\mathbf{rA_3T_3 \mid G_4C_2}$	-3617.079288	12.05	-3616.948738	12.39	5.46	-0.2609	-0.2806	-0.0183	-0.0140
	$\mathbf{G_4C_2 \mid A_5T_1}$	-3541.891231	14.38	-3541.759569	14.47	0.93	-0.2628	-0.2843	-0.0202	-0.0129
**SSB-R-DNA**	$\mathbf{A_1T_5 \mid G_2C_4}$	-3541.875825	9.60	-3541.748548	9.90	4.42	-0.2595	-0.2751	-0.0224	-0.0175
	$\mathbf{G_2C_4 \mid rA_3T_3}$	-4108.281099	11.63	-4108.15256	11.54	3.29	-0.2618	-0.2842	-0.0235	-0.0199
	$\mathbf{rA_3T_3 \mid G_4C_2}$	-4108.269588	6.97	-4108.141662	7.34	2.19	-0.2560	-0.2853	-0.0252	-0.0177
	$\mathbf{G_4C_2 \mid A_5T_1}$	-3541.873028	8.01	-3541.744554	8.16	4.73	-0.2572	-0.2787	-0.0241	-0.0169

Table S5. Energies: Ground (EGR) and Excitation (EEX) state energies and Excitation and HOMO Energies as well as corresponding Dipole Moments Ground, Excitation, and Transition (DMG, DMEX, D12) in Debays of distal base pairs extracted from selected trimers of ds-oligonucleotides, calculated at the M06-2x/6-31++G** level of theory in the aqueous phase using the DFT or TD-DFT methodology.

SYSTEM	B.P. Dimer	E^{GR}	DM^{GR}	E^{EX}	DM^{EX}	D_{12}	E^{HOMO}	E^{HOMO-1}	E^{LUMO}	E^{LUMO+1}
R-DNA	A_1T_5 | rA_3T_3	-1842.384196	3.75	-1842.250287	3.99	9.58	-0.2825	-0.2850	-0.0189	-0.0123
	G_2C_4 | G_4C_2	-1874.505972	13.01	-1874.364911	12.33	4.94	-0.2658	-0.2665	-0.0179	-0.0141
	rA_3T_3 | A_5T_1	-1842.384619	4.07	-1842.251168	4.12	9.47	-0.2825	-0.2871	-0.0187	-0.0130
RE-R-DNA	A_1T_5 | rA_3T_3	-1842.384446	3.62	-1842.250413	4.07	9.81	-0.2837	-0.2843	-0.0187	-0.0121
	G_2C_4 | G_4C_2	-1874.505089	13.14	-1874.364155	13.02	4.92	-0.2656	-0.2657	-0.0177	-0.0142
	rA_3T_3 | A_5T_1	-1842.38496	3.42	-1842.251454	3.51	8.71	-0.2844	-0.2864	-0.0185	-0.0129
IM-R-DNA	A_1T_5 | rA_3T_3	-1842.384541	3.78	-1842.384541	4.22	9.82	-0.2830	-0.2836	-0.0187	-0.0122
	G_2C_4 | G_4C_2	-1874.50516	13.94	-1874.50516	13.80	5.15	-0.2657	-0.2661	-0.0179	-0.0145
	rA_3T_3 | A_5T_1	-1842.384464	3.88	-1842.384464	3.94	10.23	-0.2657	-0.2661	-0.0179	-0.0145
SSB-R-DNA	A_1T_5 | rA_3T_3	-1842.369772	3.48	-1842.369772	3.88	8.59	-0.2847	-0.2861	-0.0198	-0.0168
	G_2C_4 | G_4C_2	-1874.49108	13.00	-1874.356009	11.34	5.78	-0.2636	-0.2666	-0.0204	-0.0193
	rA_3T_3 | A_5T_1	-1842.369772	3.48	-1842.240365	3.17	8.25	-0.2844	-0.2860	-0.0201	-0.0171

Table S6. The energies (in Hartree) of Neutral, Vertical Neutral Cation, Vertical Neutral Anion forms of Base Pairs (BP) extracted from ds-oligonucleotides calculated at the M06-2x/6-31++G** level of theory in the aqueous phase, taken for stacking interaction energy calculations.

	**Neural**			**Vertical Neutral Cation**			**Vertical Neutral Anion**		
**DNA**	**Dimmer**	**Upper BP**	**Botom BP**	**Dimmer**	**Upper BP**	**Botom BP**	**Dimmer**	**Upper BP**	**Botom BP**
**A_1T_5 ∣ G_2C_4**	-1858.467934	-921.191682	-937.252595	-1858.467934	-921.191682	-937.252595	-1858.468592	-921.191767	-937.25153
**G_2C_4 ∣ A_3T_3**	-1858.468344	-937.252595	-921.191961	-1858.468344	-937.252595	-921.191961	-1858.466626	-937.25153	-921.192473
**A_3T_3 ∣ G_4C_2**	-1858.467944	-921.192489	-937.252759	-1858.467944	-921.192489	-937.252759	-1858.446669	-921.192473	-937.233582
**G_4C_2 ∣ A_5T_1**	-1858.468728	-937.252759	-921.191961	-1858.468728	-937.252759	-921.191961	-1858.449153	-937.233582	-921.190968
**A_1T_5 ∣ A_3T_3**	-1842.384901	-921.191682	-921.192489	-1842.384901	-921.191682	-921.192489	-1842.384996	-921.191767	-921.192473
**G_2C_4 ∣ G_4C_2**	-1874.50602	-937.252595	-937.252759	-1874.50602	-937.252595	-937.252759	-1874.48568	-937.25153	-937.233582
**A_3T_3 ∣ A_5T_1**	-1842.385208	-921.192489	-921.191961	-1842.385208	-921.192489	-921.191961	-1842.384127	-921.192473	-921.190968
**R-DNA**									
**A_1T_5 ∣ G_2C_4**	-1858.467503	-921.191584	-937.252337	-1858.439554	-921.184576	-937.234224	-1858.467406	-921.191514	-937.252032
**G_2C_4 ∣ rA_3T_3**	-1858.465508	-937.252337	-921.191886	-1858.440801	-937.234224	-921.18427	-1858.462316	-937.252032	-921.189807
**rA_3T_3 ∣ G_4C_2**	-1858.465603	-921.191886	-937.252897	-1858.450428	-921.18427	-937.245568	-1858.443187	-921.189807	-937.231508
**G_4C_2 ∣ A_5T_1**	-1858.470188	-937.252897	-921.191927	-1858.454594	-937.245568	-921.185217	-1858.448086	-937.231508	-921.190962
**A_1T_5 ∣ rA_3T_3**	-1842.384196	-921.191584	-921.191886	-1842.369654	-921.184576	-921.18427	-1842.382068	-921.191514	-921.189807
**G_2C_4 ∣ G_4C_2**	-1874.505972	-937.252337	-937.252897	-1874.480564	-937.234224	-937.245568	-1874.484141	-937.252032	-937.231508
**rA_3T_3 ∣ A_5T_1**	-1842.384619	-921.191886	-921.191927	-1842.370334	-921.18427	-921.185217	-1842.381614	-921.189807	-921.190962
**RE-R-RNA**									
**A_1T_5 ∣ G_2C_4**	-1858.468046	-921.19186	-937.252134	-1858.467219	-921.191654	-937.25139	-1858.467715	-921.191411	-937.25184
**G_2C_4 ∣ rA_3T_3**	-1858.466837	-937.252134	-921.191847	-1858.46604	-937.25139	-921.191244	-1858.46502	-937.25184	-921.191734
**rA_3T_3 ∣ G_4C_2**	-1858.465164	-921.191847	-937.252338	-1858.435235	-921.191244	-937.226477	-1858.426781	-921.191734	-937.217711
**G_4C_2 ∣ A_5T_1**	-1858.469281	-937.252338	-921.192285	-1858.441084	-937.226477	-921.192437	-1858.432912	-937.217711	-921.191026
**A_1T_5 ∣ rA_3T_3**	-1842.384446	-921.19186	-921.191847	-1842.38371	-921.191654	-921.191244	-1842.383913	-921.191411	-921.191734
**G_2C_4 ∣ G_4C_2**	-1874.505089	-937.252134	-937.252338	-1874.478524	-937.25139	-937.226477	-1874.470107	-937.25184	-937.217711
**rA_3T_3 ∣ A_5T_1**	-1842.38496	-921.191847	-921.192285	-1842.384497	-921.191244	-921.192437	-1842.383429	-921.191734	-921.191026
**IM-R-DNA**									
**A_1T_5 ∣ G_2C_4**	-1858.468542	-921.191814	-937.25205	-1858.456168	-921.191651	-937.241537	-1858.467688	-921.191454	-937.251865

**$G_2C_4 \mid rA_3T_3$**	-1858.465683	-937.25205	-921.191997	-1858.451321	-937.241537	-921.19142	-1858.464924	-937.251865	-921.191817
**$rA_3T_3 \mid G_4C_2$**	-1858.466806	-921.191997	-937.252412	-1858.467631	-921.19142	-937.252799	-1858.444996	-921.191817	-937.233428
**$G_4C_2 \mid A_5T_1$**	-1858.468608	-937.252412	-921.191681	-1858.46993	-937.252799	-921.191905	-1858.449176	-937.233428	-921.190464
**$A_1T_5 \mid rA_3T_3$**	-1842.384541	-921.191814	-921.191997	-1842.383853	-921.191651	-921.19142	-1842.384038	-921.191454	-921.191817
**$G_2C_4 \mid G_4C_2$**	-1874.50516	-937.25205	-937.252412	-1874.49518	-937.241537	-937.252799	-1874.485847	-937.251865	-937.233428
**$rA_3T_3 \mid A_5T_1$**	-1842.384464	-921.191997	-921.191681	-1842.384179	-921.19142	-921.191905	-1842.382946	-921.191817	-921.190464
**SSB-R-DNA**									
**$A_1T_5 \mid G_2C_4$**	-1858.453954	-921.184743	-937.245941	-1858.45518	-921.191539	-937.241743	-1858.465776	-921.191606	-937.250397
**$G_2C_4 \mid rA_3T_3$**	-1858.453814	-937.245941	-921.184277	-1858.462826	-937.241743	-921.191345	-1858.446687	-937.250397	-921.17379
**$rA_3T_3 \mid G_4C_2$**	-1858.448143	-921.184277	-937.244253	-1858.462826	-921.191345	-937.252556	-1858.442802	-921.17379	-937.249116
**$G_4C_2 \mid A_5T_1$**	-1858.453806	-937.244253	-921.18524	-1858.470381	-937.252556	-921.192024	-1858.464428	-937.249116	-921.191678
**$A_1T_5 \mid rA_3T_3$**	-1842.369772	-921.184743	-921.184277	-1842.383769	-921.191539	-921.191345	-1842.366093	-921.191606	-921.17379
**$G_2C_4 \mid G_4C_2$**	-1874.49108	-937.245941	-937.244253	-1874.495195	-937.241743	-937.252556	-1874.500163	-937.250397	-937.249116
**$rA_3T_3 \mid A_5T_1$**	-1842.370483	-921.184277	-921.18524	-1842.38438	-921.191345	-921.192024	-1842.366292	-921.17379	-921.191678

Table S7. The energies (in Hartree) of Neutral forms of Nucleoside Pairs (NP) extracted from ds-oligonucleotides calculated at the M06-2x/6-31++G** level of theory in the aqueous phase, taken for stacking interaction energy calculations. Zip file (PDB Structures.zip) of the optimized PDB structure of the discussed molecules' structures.

	**DNA**			**R-DNA**			**RE-R-DNA**		
	**Dimmer**	**Upper BP**	**Botom BP**	**Dimmer**	**Upper BP**	**Botom BP**	**Dimmer**	**Upper BP**	**Botom BP**
**$A_1T_5 \mid G_2C_4$**	-3541.894048	-1762.893134	-1778.958352	-3541.892494	-1762.890521	-1778.957489	-3541.897146	-1778.959669	-1762.893195
**$G_2C_4 \mid A_3T_3$**	-3541.896894	-1778.958352	-1762.898242	-3617.09209	-1778.957489	-1838.096648	-3541.892498	-1762.890753	-1778.956932
**$A_3T_3 \mid G_4C_2$**	-3541.894808	-1762.898242	-1778.957372	-3617.096342	-1838.096648	-1778.959669	-3617.091646	-1778.956932	-1838.094865
**$G_4C_2 \mid A_5T_1$**	-3541.892381	-1778.957372	-1762.892175	-3541.897146	-1778.959669	-1762.893195	-3617.075178	-1838.094865	-1778.95191
	**IM-R-DNA**			**SSB-R-DNA**					
**$A_1T_5 \mid G_2C_4$**	-3541.893251	-1762.891187	-1778.956821	-3541.875825	-1762.884835	-1778.947616			
**$G_2C_4 \mid rA_3T_3$**	-3617.087914	-1778.956821	-1838.093702	-4108.281099	-1778.947616	-2329.289188			
**$rA_3T_3 \mid G_4C_2$**	-3617.079288	-1838.093702	-1778.955869	-4108.269588	-2329.289188	-1778.951211			
**$G_4C_2 \mid A_5T_1$**	-3541.891231	-1778.955869	-1762.892576	-3541.873028	-1778.951211	-1762.885483			
